# The validity of Blue Zones demography: a response to critiques

**DOI:** 10.1093/geront/gnaf246

**Published:** 2025-12-17

**Authors:** Steven N Austad, Giovanni M Pes

**Affiliations:** Department of Biology, University of Alabama at Birmingham, Birmingham, Alabama, United States; Department of Medicine, Surgery and Pharmacy, University of Sassari, Sassari, Italy

**Keywords:** Age verification, Exceptional longevity, Defined geographic areas

## Abstract

Blue Zones are geographically and temporally defined areas with a history of disproportionately high concentrations of nonagenarians and centenarians. Nearly two decades ago, these zones gained international attention when the Blue Zone term was introduced in seminal articles published in *Experimental Gerontology* and *National Geographic*. Since then, numerous scientific papers have extracted valuable insights into human health from investigating the long-lived people who live there. However recently, validity of the ages of people living in the Blue Zones has been questioned. Here, we address these concerns by describing in detail the age validation process undertaken in Blue Zones and comparing it to the prevailing standards in gerontological demography. As discovered a century and a half ago, *most* self-reported claims of exceptional longevity are false. However, using methods developed by gerontological demographers over the past decades, the true age of people claiming exceptional longevity can be determined by cross-checking multiple independent documentary sources. This procedure minimizes, and usually eliminates, errors due to fraud, honest mistakes, poor memory, or identity switches, especially between homonymous siblings. All Blue Zones described herein have been extensively validated based on thoroughly cross-checked data from multiple independent sources plus state-of-the-art demographic methods. Consequently, age data from these Blue Zones are valid and reliable.

People and populations claiming exceptional longevity have been a subject of fascination since ancient times. The Bible narrates the story of outlandishly long-lived patriarchs surviving many centuries. Mention of exceptional longevity is also found in the ancient Roman world in the writings of Pliny the Elder, Valerius Maximus, and Phlegon of Tralles (Floris et al., 2021) continuing with similar credulous accounts related by some of the leading scholars of the17th and 18th centuries such as Francis Bacon and John Locke (Laslett, 1999). There arose (and continues) what historian Peter Laslett called a “cult of centenarians.” However, beginning in the second half of the 19th century, skepticism about extreme age claims emerged and such claims began to be subjected to evidentiary scrutiny (Thoms, 1873, 1879; Young, 1899). In the 20th century, as more and more demographers and actuaries examined claims of extreme longevity, they noticed that even official government records were often fraught with errors, typically vastly overstating survival to later life (Kannisto, 1988, 1994). For instance, according to official records someone was more than twice as likely to live to 100 if she lived in Argentina, Bulgaria, Ireland, the Philippines, or Russia in 1900 than if she lived in 1990 Japan, then as now the longest-lived country in the world (Austad, 1997). Consistent with this pattern, as literacy improved alongside better and better record keeping, the number of official centenarians fell in country after country even as life expectancy in those countries increased. Thanks to these demographic efforts, validating the ages of our oldest people has become something of a demographic specialty (Jeune & Vaupel, 1999; Kannisto, 1994; Manton & Vaupel, 1995; Rosenwaike & Preston, 1984), we now know that most self-reported claims of extreme longevity are false. That does not mean, however, that exceptionally old people and populations do not exist. It only means that identifying them is not a trivial task.

Thoms in the 19th century proposed the following steps for investigating claims of exceptional longevity (Poulain, 2010): (i) search for official birth records; (ii) check the records for other individuals with the same name; (iii) corroborate the birth record with additional forms of evidence; and (iv) if the person is still alive, verify as much as possible by quizzing the person about things such as public events that can be cross-checked with additional data sources. Modern gerontological demographers still basically follow Thoms’ procedure but now there are many more public data sources available for cross-checking.

People unfamiliar with these long-standing demographic issues can still be fooled by false claims, however, especially about something they wish to believe. Broad interest in geographic areas with extreme longevity was kindled in the 1960s by several widely viewed sources such as a 1966 article in *Life* magazine on people living in a village in current-day Azerbaijan reported that the villagers regularly reached ages topping 150 years (Young, 1966). A *60 Minutes television* segment on exceptional longevity in the same village ran a decade later. But what really started a new cult of longevity was when *National Geographic* magazine sent distinguished Harvard physician Alexander Leaf to reputed longevity hotspots in the remote mountains of Ecuador, Pakistan, and today’s Azerbaijan, from which he reported back credulously on seemingly any ages the local residents told him (Leaf, 1973). Distinguished as he may have been, Dr. Leaf was alas innocent of the long history of age exaggeration as well as the techniques that had been developed to detect it. Leaf’s story was fairly quickly invalidated by people more familiar with the culture of age exaggeration and better trained to detect it (Mazess & Forman, 1979; Medvedev, 1974). The inhabitants of these areas, it turned out, had falsified their age either for commercial gain, to elude the military draft, or possibly just for the enjoyment of foxing an outsider.

Alexander Leaf’s debunked story had a sobering influence on professional gerontologists, who, for a time, became skeptical about the existence of any long-lived populations, and largely abandoned this line of research. But the emergence of a significant proportion of the oldest old in resource-rich ­countries and some developing countries has forced researchers to reconsider the study of the exceptionally long-lived populations.

*When one of us (G.M.P.*) in 1999 presented for the first time data suggesting the existence of exceptionally long-lived communities in the central area of the Mediterranean island of Sardinia, where the proportion of centenarians was demonstrably higher than in the rest of the island (Pes, 1999), most demographers reacted with skepticism. From 2001 to 2019, a collaboration with the Belgian demographer Michel Poulain was able to demonstrate that Sardinian longevity in these areas was genuine. For instance, it was determined that people born in this longevity hotspot between 1880 and 1900 were nearly three times as likely to live to age of 100 years as Sardinians living outside the area. The area was dubbed a Blue Zone because of the blue marker used to indicate the area on a map. The first scientific article mentioning this term was published in *Experimental Gerontology* in 2004 (Poulain et al., 2004).

Since 2005, the concept of Blue Zone was extended to other regions of the globe in which exceptional longevity similar to the Sardinian one was found (Buettner, 2008). Importantly, these areas were not only characterized by their exceptional number of older people but also by the exceptional health and vigor of these people. This prolonged health is an important observation, particularly at a time when later life morbidity seems to be expanding in many parts of the world (Rowe, 2024).

Using strict demographic criteria, exceptionally long-lived communities have been validated in four locations: Okinawa, Japan; Sardinia, Italy; Ikaria, Greece; and Nicoya, Costa Rica. Following these discoveries, the international scientific community generally accepted the Blue Zone concept (Appel, 2008; Buettner & Skemp, 2016; Huang & Mark Jacquez, 2017; Liu et al., 2020; Pes et al., 2022; Poulain et al., 2021).

However, even now researchers from outside demographic gerontology, and unfamiliar with the history and practice of age validation, continue to question these long-validated results, conflating them with real age misinformation. Two recent examples are (i) an attempt to invalidate the age of Jeanne Calment, the oldest woman on record (Zak, 2019), who ironically is also one of the best validated supercentenarians ever with at least 30 pieces of confirming documentation (Robine et al., 2019); (ii) a critique challenging a broad swathe of extreme longevity claims including the authenticity of Blue Zones (Newman, 2024a, 2024b). Because these critiques have captured the attention of popular media, as researchers with an abiding interest in validation of both human and animal extreme age claims (Austad, 1997, 2022; Pes, 2023; Poulain et al., 2006) and as a Blue Zone discoverer (G.M.P.), we felt it necessary to provide an overview of the age validation process used in identifying Blue Zones and compare it to the standards of practice in gerontological demography science. Validation of the Blue Zones, we believe, is important because there are valuable lessons to be learned, and inspiration to be gathered, from lifestyles that facilitate long, healthy lives. Documenting the disappearance of Blue Zones can be equally informative about lifestyle factors associated with worse later life health.

## Standards of practice in demography

The thorough age validation approach adopted for Blue Zones, hinging on multiple independent data sources, has minimized the risk of false claims and ensures that cases of exceptional longevity are credible and well-documented. Civil records, such as birth and death certificates, marriage records, military records, and electoral registers, have been cross-checked with church or family archives to confirm consistency in birth dates, names, and family history. Genealogical reconstruction was frequently used to identify discrepancies, such as reused names after sibling deaths, and ensures that identities are not confused. In addition, personal interviews with exceptionally old people and their families provide additional confirmation of life events and consistency with official records. Advanced data systems and unique identification numbers make forgery much less likely than previously. Historical records are also reconstructed where necessary. During the validation procedures, cases where discrepancies cannot be resolved are rejected. Especially in Sardinia and Okinawa, rare but real documented cases of false age claims have been discovered and were systematically removed from Blue Zone databases.

## Details of age verification in each Blue Zone

Blue Zone attribution requires that a place be geographically defined, possess an exceptionally high concentration of people attaining the age of 90 years or over in the past 150 years, and has records that can validate the birth and death dates of the cohorts or provide proof of life status for those over the age of 90 years. It is important to emphasize that Blue Zones have never been about the number of supercentenarians (those 110 years old or older) in an area, but instead is about the relative proportion of a population aged 90 years or older. It should also be noted that Blue Zones are not necessarily permanent. Modernization, westernization, as well as immigration and emigration may alter or dilute local health and longevity such that a Blue Zone may disappear or a new one appear. Indeed, due to westernization Okinawa, as noted below, no longer qualifies as a Blue Zone (Poulain & Herm, 2024) and the Nicoya Blue Zone is shrinking due to later born men living shorter lives for unclear reasons than those born earlier (Rosero-Bixby, 2023).

The age verification process requires (i) identifying potential high-longevity target areas based on public demographic databases; (ii) accurately counting the number of people born in the target area, categorized by sex and year of birth; (iii) identifying and confirming the age, using additional data sources, of individuals who have reached a preset threshold age (e.g., 90 years); and (iv) calculating the ratio of the 90+ population to total births recorded during the same time interval. This ratio reflects the probability of people born in the target area to reach the threshold age. The process is designed to minimize the likelihood of false positives (type I errors) and ensure accurate identification of Blue Zones. In the following, detailed age verification procedures in each of the four original Blue Zones is described.

### Sardinia, Italy Blue Zone validation

As previously mentioned, the Sardinian Blue Zone was first described because of its high percentageof centenarians (0.51%) among the cohorts born between 1880 and 1900. This Blue Zone was centered on six villages located in the hills of a rural region in east central Sardinia called Ogliastra containing about 12,000 people. This percentage was approximately five times as high as the percentage of centenarians throughout Europe and nearly three times the percentage in Sardinia as a whole. Equally surprising*, although worldwide* there are roughly three times as many female as male centenarians—in the Sardinian Blue Zone there were approximately equal numbers of male and female centenarians (Poulain et al., 2004). Since that original analysis, the proportion of centenarians in this Blue Zone has increased (Poulain & Herm, 2025).

The age validation process does not rely on self-declaration only but instead on written records from multiple independent sources. Specifically in the case of Sardinia, the age of every nonagenarian or centenarian was double-checked using (i) civil status databases dating back to 1866, (ii) handwritten records from ecclesiastical archives, consistently available from the 17th century onward, and (iii) complete genealogical reconstruction of village inhabitants from 1866 onward (Poulain et al., 2006). Throughout the validation process, the compatibility of these various documentary sources was carefully ascertained, including the consistency of name and surname of each individual, their relatives, the marriage date of the parents, and the plausibility of their age at marriage (Poulain et al., 2006). Ken Wachter, a noted demographer at the University of California, Berkeley, observed that birth certificates in Italy, including Sardinia, are very accurate and “filled out by Civil Status Officers, well educated for their time, who entered births chronologically into bound volumes, a separate volume for each year in each municipality” (Wachter, 2018).

In addition to civil registers, ecclesiastical certification (*Quinque Libri* of the Catholic Church) has systematically been used in the villages with the highest concentration of longevous people. For each candidate 90 years or older—hereafter called the “proband”—any discrepancy between civil and ecclesiastical records was ruled out. Importantly, this procedure did identify an alleged Sardinian female supercentenarian whose death record was incompatible with her birth record due to an identity switch with an older sister. That case was duly published (Poulain et al., 2006). Thus, double or triple verification ensures that the probability of errors in the proband’s birthdate is virtually zero. Additional information, such as date of enrolment in the army, admission to school, or public employment, can be a valuable complement to civil and ecclesiastical documents.

Through both civil and ecclesiastical archives, the analysis of probands’ genealogy also included their siblings. All brothers and sisters’ birth and death records were checked to verify the length of birth spacing (i.e., interpregnancy and interbirth interval). In the Sardinia Blue Zone, it was not unusual for parents to give a newborn the same name of a sibling who had died shortly before. Thus, birth intervals between siblings were analyzed during the validation process to identify any incompatibility or homonymy cases and rule out stolen identities. It was the careful family reconstruction that allowed identification of the inaccuracy mentioned above. The person who had died at the presumed age of 110 years was actually the proband’s younger sister. She had been given the same name but was born 3 years later. This false supercentenarian was duly eliminated from the list of Sardinian supercentenarians (Poulain et al., 2006). Similarly, using ecclesiastical archives, it was possible to invalidate the age of another alleged Sardinian supercentenarian born in the 19th century (Pes, 2023). The accuracy of an older person’s age can fall into various levels, first proposed by Skytte and Jeune (1995): (i) Level D, date and age at death without verification; (ii) Level C, birth registration; (iii) Level B, life story in addition to evidence from Level C; and (iv) Level A, family reconstructed in addition to the evidence from Level B. In Sardinia, the maximum level of verification, Level A, has been reached regarding the six villages forming the Blue Zone. Therefore, the entire genealogy of each village has been sifted through to exclude any erroneous identity attribution.

Despite some modernization on the island, the Sardinian Blue Zone remains intact (Poulain & Herm, 2025).

### Nicoya, Costa Rica Blue Zone validation

The Nicoya Blue Zone consists of five neighboring cantons: Santa Cruz, Carrillo, Nicoya, Nandayure, and Hojancha in the northern part of the Nicoya Peninsula, Costa Rica. Exceptional longevity is found only in men of the Nicoya Blue Zone, specifically men who were born before 1930. Examination of these men during the period 1990–2011found that 60–69 years old men were seven times as likely to reach the age of 100 years as Japanese males of approximately the same time period—a time at which Japan was the longest-lived country in the world (Rosero-Bixby et al., 2013).

Validation of the oldest old in the Nicoya Peninsula was performed by observing consistent ages in three independent databases. One is a birth registry which has been in uninterrupted existence since 1883. In that registry at birth or naturalization, each Costa Rican receives an identification number for life. That number is on the person’s national identification card (the *cédula*), which also has the birth date. Individuals registered years after their birth due to naturalization or a missing birth record are therefore identifiable and excluded from the analyses. Birth ledgers are sequentially numbered within each of the seven Costa Rican provinces, as well as within the pages within each book and the lines within each page. The identification number of a Costa Rican includes the province code, the volume, the page, and the starting line where their birth (or naturalization) is entered. Birth ledgers are kept in secure government (TSE) vaults. Second, age is confirmed using the public electoral list, known as *padrón electoral*, which is published by the National Electoral Tribunal (*Tribunal Supremo Electoral* or TSE), which dates back to 1949 and is in charge of the birth registry. These electoral registers are detailed records of each person who is entitled to vote and also include relevant information used to identify voters and assign them to a specific electoral district and a voting center. The TSE includes birth and death data for all living Costa Ricans who have contacted the civil registration system since its computerization in 1970 (Rosero-Bixby, 2008; Rosero-Bixby et al., 2013). Final confirmation used the death registry itself. Recent demographic evaluations conclude that the Costa Rican death registration system is essentially free of under-registration errors (INEC & CCP, 2013). Since 1961, the United Nations has considered the Costa Rican vital statistics system as “complete” (United Nations, 1961). Also, the civil status data can be compared with similar data in the ecclesiastical archives.

Additionally, in 2007, one of us (G.M.P.) along with Dan Buettner and Michel Poulain visited villages on the Nicoya Blue Zone to verify the age of 35 putative centenarians. We crosschecked birth dates on their *cédulas* against the birth registers in their respective municipalities. In doing so, we were able to invalidate the age of one of the 35, who claimed to be 107 years old, but in fact had not yet become a centenarian. He was duly eliminated from the Nicoya database.

We should note that by 2010, new analyses found the original Nicoya Blue Zone had shrunk to about one-quarter its original size but surprisingly, a new Blue Zone had emerged in three provinces in northern Costa Rica near the Nicaraguan border (Rosero-Bixby, 2023). Investigation of changes in lifestyle factors as well as public health and medical changes in both of these areas will be illuminating.

### Ikaria, Greece Blue Zone validation

Ikaria Island, Greece, a small, mountainous island with a population of about 8,000 people in the eastern Aegean Sea, was first described as a Blue Zone in 2009 when it was discovered that the proportion of inhabitants aged 90 years and older was approximately three times higher than the Greek national average (Poulain & Pes, 2009).

The validation of the age of the oldest-old inhabitants of Ikaria, who largely lacked birth records, was conducted by comparing the mortality statistics provided by the Greek central institutes with the data extracted from the “*dimotologion*” (Δημοτολόγιο), an administrative registry (Poulain & Pes, 2009) that includes demographic information on all Greek citizens of a given municipality. Individual age validation was also successfully achieved during exhaustive interviews with all those aged 90 and above on the island by using a battery of questions on the occurrence of demographic events and the age of close relatives (Poulain & Pes, 2009).

The validation of exceptional longevity on Ikaria Island was conducted between 2008 and 2009 by consulting multiple sources: (i) the Hellenic Statistical Authority (Greek: ΕΛΣΤΑΤ, Elstat), the primary demographic institution in Greece; (ii) the prefecture of Karlovasi, Samos Island, to which Ikaria administratively belonged; and (iii) the offices of the three main municipalities on Ikaria: Raches (Ράχες), Evdilos (Εύδηλος), and Agios Kirykos (Άγιος Κήρυκος).

Censuses in Greece were established in 1838 and from 1839 onward results were published in the Official Journal. These censuses distinguished among the *de facto* population (πραγματικός; individuals present during the census, irrespective of permanent residence), the permanent population (μόνιμος; individuals declaring the area as their permanent residence), and the legal population (νόμιμος; citizens permanently residing in the municipality). The difference between legal and permanent populations allows for an estimate of the number of foreign residents.

During the validation process, Elstat provided data on deaths, disaggregated by municipality, age, and sex, for the period 1995–2006. The prefecture of Samos supplied 2001 census data for Ikaria, categorized into the three aforementioned population types. A comparison of these data with national figures revealed that the proportion of individuals aged 90 years and older on Ikaria was approximately three times higher than the Greek national average.

Additionally, access to municipal birth registers—available from 1890 and complete from 1913 (the year Ikaria was annexed to Greece following Ottoman rule)—allowed further validation. Data collected from both the Ikaria municipalities and Karlovasi provided the following: (i) the number and lists of deceased individuals aged 90 years and older and (ii) the number and lists of living nonagenarians and centenarians. In 2009, no fewer than 124 individuals aged 90+ were confirmed to be living on the island, corresponding to a prevalence of 1.49%, nearly five times the prevalence (0.33%) on the Greek mainland. Furthermore, of approximately 800 deaths recorded on Ikaria between 1900 and 2006, around 200 occurred among individuals aged 90 years or older, including 20 centenarians. This represented a 2.5% percentage of centenarians, notably higher than that observed in the Sardinian Blue Zone (2% at that time). Despite some inconsistencies in census and mortality data completeness, the findings clearly indicated that longevity on Ikaria exceeded that of Greece overall. The proportion of 90-year-olds was markedly higher, and the distribution of deaths by age demonstrated a significant shift toward older ages.

### Okinawa, Japan Blue Zone validation

Okinawa is a small island southwest of the four major Japanese islands. In 1976, Kagawa and colleagues reported that the percentage of centenarians on Okinawa was nearly seven times as high as in the rest of Japan (Poulain & Herm, 2024). Between 1972 and 2006, the Japan Annual Centenarian Report ranked Okinawa the highest of the 47 Japanese prefectures for prevalence of centenarians, although by 2006 its centenarian rate was only about twice that of the rest of Japan (Willcox et al., 2008). The Okinawan Centenarian Study (OCS) began in 1975 and has continued since (Willcox et al., 2008). The sources used for age validation were (i) The National Directory of the Elderly (全国高齢者名簿) published by the Ministry of Health, Labor and Welfare and available for Okinawa since 1975; (ii) the ofﬁcial statistics from population censuses and vital registration issued since 1920; and (iii) regional life tables for each of the 47 prefectures, including Okinawa (Poulain & Herm, 2024). Though many birth records were destroyed during WWII bombings, 20 copies of the Okinawan birth records had been preserved and microfilmed, such that the documents lost during wartime have been reconstructed. In addition to these sources, investigators from the OCS visited the residences of 100 of study participants, gathering verbal verification from the centenarian themselves, such as consistency between the stated birth year and its associated Chinese Zodiac symbol, something important in Okinawan culture. Investigators also examined other documents provided by the centenarians, such as medical and school records, military certificates, and any other documents that listed the centenarian’s age. They performed family reconstructions as well, including birth dates of the centenarian’s parents, siblings, children, and grandchildren to confirm plausibility of the documented age. In 2005, Willcox and colleagues examined the records of an additional sample of 52 centenarians in three Okinawa municipalities and found a perfect match with self-reported information in 49 of them (94.2%). Only in three cases was the stated age more than one year different from the documented age. No evidence of a systematic age exaggeration was found (Willcox et al., 2008).

In 2010, it was widely reported in the world press that 230,000 Japanese centenarians had gone “missing.” The story followed the discovery of the corpse of a man who would have been 111 years old if alive, but who had apparently died several decades earlier. His death had never been officially reported as his surviving kin continued to receive his pension. The 230,000 figure was due to inconsistencies between two independent national registries in whether someone had died or not. That news report led some demographers to question exceptional Japanese longevity but also led local officials to urgently seek proof-of-life for centenarians in their jurisdiction usually by visiting their residence or meeting them in person. These verification records turned up about 600 actually missing centenarians across the 47 Japanese prefectures. Importantly, however, zero missing centenarians turned up in Okinawa (Saito et al., 2012).

## Discussion

We have shown how extensively validated have been the ages of the people and places that defined the originally described Blue Zones of the world. We did this because we found it important to counter recent claims that they represented little more than areas of poor demographic record-keeping or rampant age-exaggeration. These claims in fact called into question the existence of Blue Zones. In presenting this extensive age validation, we have also noted that Blue Zones are not forever—old ones may disappear, new ones appear. A challenge for researchers is to discover the reasons for these changes.

A common theme of these four classic Blue Zones is isolation. Three of the four (Sardinia, Ikaria, Okinawa) occupy all or part of islands, the fourth (Nicoya) lies on a peninsula that until recent times was difficult to access. The relative isolation of the islands is apparent in that each has evolved its own language or dialect relative to the mainland. Isolation makes possible cultural, and possibly genetic, uniqueness. Other than isolation there is little geographic, climatic, or ecological similarity among the Blue Zones. Ikaria and Sardinia are rocky and mountainous, Nicoya is mountainous but dominated until recently by tropic forest, and Okinawa is relatively flat.

A recent critique of *the existence of* Blue Zones noted that they often occur in areas that are economically deprived and have high crime rates. In the modern developed world, people living in such areas tend to be short-lived rather than long-lived. It is true that a certain degree of economic underdevelopment has historically characterized the Blue Zones. This should be no surprise as Blue Zones are defined by being exceptional. The cases of Okinawa and Sardinia clearly indicate that exceptional longevity is not inevitably incompatible with economic deprivation (Cockerham et al., 2000).

A large body of evidence supports the conclusion that high crime rates are part of cocktail of factors underlying the social determinants of health (Chetty et al., 2016; Nosrati et al., 2018). These social determinants are typically associated with low life expectancy. However, it is important not to conflate current conditions with the conditions in which the oldest people existed for most of their lives. It is also important not to conflate crime in a large region with that in a smaller Blue Zone. For instance, in the case of the Sardinian Blue Zone, while there may be relatively high crime in Sardinia’s cities by Italian standards, that is not true in the Blue Zone villages. These six villages located in the hills of a rural region called Ogliastra contain a total of about 12,000 people compared with the total Sardinian population of 1.6 million. As is common throughout the world, crime tends to be higher in urban compared with rural areas, especially in small towns where everyone knows almost everyone else (Glaeser & Sacerdote, 1999). However, even in Sardinia as a whole the homicide rate in 2021 was only 0.6 per 100,000 people (https://oecdregionalwellbeing.org/ITG2.html), which is somewhat lower than the European Union rate of 0.86 per 100,000 people according to Eurostat Crime Statistics, 2025 and (Ventura et al., 2022). In the Blue Zone villages, it is substantially lower than this.

Importantly, Blue Zones do not necessarily last forever. Migration, urbanization, westernization, or other still-to-be-discovered factors may destroy *a once* thriving Blue Zone. The population of Okinawa, which in 1999 produced the world’s, well-validated longest-lived people, no longer meets Blue Zone requirements (Poulain & Herm, 2024). Cohorts born prior to 1940 defined the Okinawan Blue Zone. Since then, the ravages of war combined with massively increasing westernization brought about during the large U.S. military presence on the island, appear to be eroding islanders’ health (Todoriki et al., 2004; Willcox et al., 2008). Similarly, in the Nicoya Costa Rica Blue Zone, the long-lived area is progressively shrinking for reasons that are not entirely clear, although increased immigration and modernization are suspected. This Blue Zone was defined by men born before 1930. Men born later are less likely to reach the age of 100 years than their predecessors. But in 2008, when the first study appeared, the oldest men in the Nicoya Blue Zone region clearly enjoyed exceptional health and longevity. Intriguingly, it appears as if a new Blue Zone is emerging in a different (northern) part of Costa Rica (Rosero-Bixby, 2023). These twin developments offer an exceptional opportunity for further investigation of the factors that make a Blue Zone.

On the other hand, new Blue Zones may emerge as has recently been reported in The Netherlands (Deeg et al., 2024), Rugao, China (Huang & Mark Jacquez, 2017), and the Caribbean Island of Martinque (Poulain & Herm, 2025). Of course, each of these newly discovered Blue Zones will require *its own extensive validation* before being widely accepted as have been the original Blue Zones.

We have not discussed what factors make for a Blue Zone, something that has been extensively covered elsewhere. Briefly, one possibility is that unique genetic factors are involved. However, multiple investigations have failed to identify an excess of longevity alleles in these populations that have been identified elsewhere (Amigo, 2024; Errigo et al., 2024). This does not disprove a genetic contribution to the exceptionally long lives in the Blue Zones. The dramatically increasing ability of modern DNA analysis to identify rare but important population variants may yet identify unique genetic factors at work in some Blue Zones. However, the lack of obvious genetic factors does seem to make it more likely that lifestyle, diet, exercise, and community practices play a more important role. Blue Zones have therefore been seen as guides for lifestyle factors leading to long healthy lives. Given that the possibility of living a long life exerts a powerful popular appeal, it is not surprising that a plethora of books, popular articles, and websites have taken over this topic. Whatever factors underlie the exceptional longevity of people living in these four Blue Zones, there is no question that they truly do exhibit exceptional longevity as established by the best modern practices in gerontological demography.

Research to date has focused extensively on the healthy older people that define Blue Zones. However, the recent appreciation that Blue Zones may come and go offers the opportunity to more precisely identify those sociocultural factors that lead to the appearance or disappearance of Blue Zones. Also, for those Blue Zones which have persisted, such as in Sardinia, there is the opportunity to investigate the younger people perhaps developing new insights into the factors that may allow them to so often survive into healthy old age.

Blue Zones provide an opportunity to study salubrious lifestyle factors in depth. They also offer an opportunity to evaluate how successfully (or not) traditional healthy lifestyles can interact with modernized infrastructure, public health policies, and medical practices. In some cases, adding modern medicine and infrastructure to traditional ways of life may extend and enhance Blue Zones, perhaps even creating new ones such as in northern Costa Rica. In other cases, as in Okinawa, modernization might be fatal to a pre-existing exceptional health and longevity. Ultimately, Blue Zones stand as a beacon for global health and longevity. Embracing their lessons offers a promising path toward a longer, healthier future.

In conclusion, while exaggeration of exceptional longevity may be rife in many parts of the world, the ages of people in these four classic Blue Zones have been extensively validated using the best techniques of modern demography. They still have much to teach the world about how to live a long, healthy life.
